# Economic Burden of Rheumatoid Arthritis in Iran: A Societal Perspective

**DOI:** 10.1002/puh2.70131

**Published:** 2025-11-12

**Authors:** Mehdi Rezaee, Farhad Lotfi, Ahmad Gholami, Jassem Azizpoor, Elham Aflaki, Afsaneh Vazin, Khosro Keshavarz

**Affiliations:** ^1^ Department of Management, Economics and Health Policy, School of Health Bushehr University of Medical Sciences Bushehr Iran; ^2^ Student Research Committee, School of Health Management and Information Sciences Shiraz University of Medical Sciences Shiraz Iran; ^3^ Health Human Resources Research Center, School of Health Management and Information Sciences Shiraz University of Medical Sciences Shiraz Iran; ^4^ Biotchnology Research Center Shiraz University of Medical Sciences Shiraz Iran; ^5^ Department of Pharmaceutical Biotechnology, School of Pharmacy Shiraz University of Medical Sciences Shiraz Iran; ^6^ Department of Rheumatology, School of Medicine Shiraz University of Medical Sciences Shiraz Iran; ^7^ Department of Clinical Pharmacy, School of Pharmacy Shiraz University of Medical Sciences Shiraz Iran

**Keywords:** cost of illness, direct medical cost, direct non‐medical costs, economic burden, indirect costs, rheumatoid arthritis

## Abstract

**Background:**

Rheumatoid arthritis (RA) is a progressive and chronic systemic inflammatory disease and imposes a significant economic burden on patients and societies if not controlled. This study aimed to determine the economic burden of RA in Iran in 2022.

**Methods:**

This economic burden study was carried out as cross‐sectional research in 2022, in which 765 patients referred to the medical centers affiliated with Shiraz University of Medical Sciences in Fars province were included through census. The prevalence‐based and bottom‐up approaches were also used, respectively, to prepare the cost information and calculate the costs from the societal perspective. The human capital approach was used to calculate the indirect costs as well. The Excel 2016 software was used for data analysis.

**Results:**

The results showed that the economic burden of RA in Iran was $6388.64 per patient‐year in 2022. In addition, the mean annual costs of RA per patient in remission, low, and moderate‐to‐severe states were, respectively, $6268.24, $6248.00, and $6729.43 in 2022.

**Conclusions:**

RA imposed a significant economic burden on the community and patients, and direct medical costs (DMCs), especially the cost of medicines, were the most important component. It is suggested to provide necessary facilities to produce RA medicines in the country and expand the home care services in order to reduce the economic burden.

## Graphical abstract

GRAPHICAL ABSTRACT

This 2022 cross‐sectional study of 765 RA patients in Southern Iran revealed an annual economic burden of $6388 per patient, with biologic drugs dominating costs (84%). Moderate‐to‐severe cases cost 8% more than remission ($6729 vs. $6248). Findings highlight the urgent need for local drug production and expanded home care services to alleviate this significant financial burden on patients and healthcare systems.

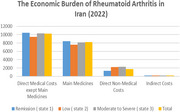

## 1 Introduction

Rheumatic diseases are chronic progressive diseases that damage the locomotor system, lead to disability, and, thus, reduce the patient's quality of life. Inflammatory rheumatic diseases are especially dangerous to health [1]. The most common inflammatory rheumatic diseases are rheumatoid arthritis (RA) and spondyloarthropathy. Progressive joint injury, pain, disability, and premature mortality are some characteristics of RA, especially if not treated in a timely manner [2]. The patients often suffer from symptoms that may be objectively difficult to quantify (e.g., fatigue), while affecting work performance, and are not often overlooked by employers [3]. In RA, the disability due to the disease is more important and frequent than mortality. Pain, dysfunction, fatigue, and depression are among the common symptoms of the disease, associated with a significant reduction in the quality of life [4].

The prevalence of RA varies in different countries. For instance, the prevalence rate of the disease in Spain and Japan in 2020 was 0.82% and 0.75%, respectively [5, 6]. In Poland, it was estimated at 0.9% in 2019 [7]. The results of a systematic review in 2020 showed that the debilitating condition could affect 0.46% of the world's population [8]. In addition, the prevalence of RA in 2016 was reported to be 0.37% in Iran [9].

On the other hand, RA is a chronic progressive autoimmune disease that imposes a significant economic burden on the patients and their families [10]. The total annual economic cost of RA in 2008 was estimated at €45.3 billion in Western Europe and €41.6 billion in the United States [11]. According to the studies conducted in recent years, the economic burden of the disease has increased significantly in such a way that the annual economic burden of RA in the United States in 2005 was $19.3 billion, of which 56% ($10.9 billion) was that of indirect costs (ICs) [12]. However, it was estimated at $48 billion in 2015 [13]. Moreover, a study conducted in Germany in 2017 showed that the direct costs of RA had increased in the last decade [14].

Considering different medical costs of RA and, thus, different financial and economic burdens on the health system, and due to the limited knowledge about its economic burden, and because the researchers could not find any studies on the economic burden of the disease in Iran, the present study was conducted to determine the economic burden of RA.

## 2 Materials and Methods

This cross‐sectional cost‐of‐illness study was carried out on RA patients who were treated with biologic or targeted systemic disease‐modifying antirheumatic drugs (tsDMARDs) and referred to the medical centers affiliated with Shiraz University of Medical Sciences in Fars province, Iran, in 2022. In this study, the prevalence‐based model was used to calculate the costs. Prevalence‐based studies deal with the total number of cases in a given period of time (usually 1 year). In this approach, the economic cost of the existing cases of the disease is estimated during a given period [15]. Therefore, all the patients with RA who had medical records and were also willing to participate in the study were included through census (*N* = 765).

### 2.1 Cost Assessment

The societal perspective was used to extract the costs in this study. From this perspective, relevant costs included direct medical costs (DMCs), direct non‐medical costs (DNMCs), and ICs.

The DMCs were collected retrospectively from January 1 to December 31, 2022, using the data collection form and by referring to the studied medical centers. The DNMCs and ICs were also collected using this data collection form and the patients’ self‐reports.

### 2.2 Direct Medical Costs

DMCs of each patient were retrospectively determined and collected by referring to the studied medical centers. For estimating the DMC, the whole disease costs were classified into six cost categories, including medicine costs (price without subsidies), physicians’ visits, laboratory tests, physiotherapy, diagnostic services, and hospitalization.

In order to increase the accuracy and precision of the data on DMCs, the subjects’ inpatient and outpatient medical records were used. In addition, to obtain an accurate analysis of the costs, the direct total costs of the medicines (price without subsidies), physicians’ visits, laboratory tests, physiotherapy, diagnostic services, and hospitalization were separately determined for the patients in 2022.

### 2.3 Direct Non‐Medical Costs

The DNMC‐related data were obtained through interviews with the patients. Because a large percentage of the patients referred to the medical centers were living out of Shiraz, items such as the cost of traveling to the centers to receive medical services, as well as accommodation and meal costs, were regarded as DNMC components for the patients and their companions.

### 2.4 Indirect Costs

The human capital approach was also used to calculate the ICs [16]. The ICs for each patient were calculated on the basis of the average daily income lost due to the patient's absence from work and sick leave for hospitalization or disease follow‐up and the average daily income lost for each companion or caregiver due to absence from work to accompany or care for the patient. In the present study, the individuals’ wages were used to calculate the lost income. In the case of the housewives and students aged 15–65 years, the daily wage determined by the Ministry of Cooperatives, Labour, and Social Welfare (according to the reports of the Labour Department in 2022, amounting to $21.08) was used as the mean daily wage [17].

To calculate the costs in this study, a bottom‐up approach was used, in which the resources used by each person were measured. Thus, this method could reveal the differences between the patients in terms of treatment [18].

It is noteworthy that in the present study, all the costs were collected on the basis of Iranian currency (Rial) and then converted into the current US dollar ($), which was equal to 42,000 Rials in Iran in 2022 for $1 [19].

Estimating the number of RA patients required the prevalence data in the country. Accordingly, the prevalence rate of the disease around the country was 0.37% in 2016 [9], and regarding the incidence and mortality rates of RA, its prevalence rate in 2022 could be estimated at 0.37%. Thus, considering the population of 83.27 million in Iran in 2022 [20], the total number of RA patients was estimated at 308,099 in the country.

Finally, after determining the prevalence rate of RA, the population, and the mean cost per patient in the present study, the economic burden of RA in Iran was calculated using the following formula [21]:

$$\begin{array}{ccc} \mathrm{Economic}\;\mathrm{Burden} & = & \mathrm{Total}\;\mathrm{cost}\;\mathrm{per}\;\mathrm{patient}\\ & & (\mathrm{DMCs}+\mathrm{DNMCs}+\mathrm{ICs})\times \mathrm{estimated}\\ & & \mathrm{number}\;\mathrm{of}\;\mathrm{patients}\;\mathrm{with}\;\mathrm{RA}\;\mathrm{in}\;\mathrm{Iran} \end{array}$$

The Excel 2016 software was used for data analysis.

### 2.5 Sensitivity Analysis

In the present study, one‐ and two‐way sensitivity analyses were done for estimating costs. In one‐way analysis and in the first step, the properties of costs were assumed constant, but the number of patients with regard to prevalence rates was considered variable. Moreover, according to the results of a study in Iran, the prevalence rate was 0.37% (95% CI: 0.29%–0.46%) [9]. Hence, 0.29% and 0.46% were considered the low and high prevalence limits, respectively. In the second step, the prevalence rates were assumed constant, but the costs were considered variable; therefore, the lower and upper limits of costs were taken into account.

Furthermore, a two‐way sensitivity analysis was done (the best‐ and worst‐case scenarios) to examine the combined effects of varying both the prevalence rate and the costs.

## 3 Results

A total of 765 patients at different stages of RA (440 patients in remission, 120 patients in low, and 205 patients in moderate‐to‐severe states) were included in this study. Table 1 summarizes the descriptive results classified according to the disease stages and based on patients’ demographic characteristics. According to Table 1, most of the patients were female (77.12%), housewives (62.09%), and not residents of Shiraz. All the patients had insurance coverage. In addition, the majority of the patients were in the age range of 45–54 (26.14%).

**TABLE 1:** Demographic characteristics of the patients studied in 2022 (*N* = 765).

Demographic characteristics		State 1 (DAS‐28<2.6) Remission		State 2 (2.6<DAS‐28<3.2) Low		State 3 (DAS‐28>3.2) Moderate‐to‐severe		Total	
		Frequency	%	Frequency	%	Frequency	%	Frequency	%
Gender	Male	125	28.41	25	20.83	5	12.20	175	22.88
	Female	315	71.59	95	79.17	180	87.80	590	77.12
	25>	5	1.14	0	0	5	2.44	10	1.31
Age (years old)	25–34	70	15.91	10	8.33	20	9.76	100	13.07
	35–44	95	21.59	45	37.50	55	26.83	195	25.49
	45–54	130	29.55	35	29.17	35	17.07	200	26.14
	55–64	120	27.27	15	12.50	50	24.39	185	24.18
	Over 65	20	4.55	15	12.50	40	19.51	75	9.80
Occupation	Employee	60	13.64	15	12.50	15	7.32	90	11.76
	Self‐employed	75	17.05	35	29.17	15	7.32	125	16.34
	Student	5	1.14	0	0	5	2.44	10	1.31
	Retired	45	10.23	5	4.17	15	7.32	65	8.50
	Housewife	255	57.95	65	54.17	155	75.61	475	62.09
	Unemployed	0	0	0	0	0	0	0	0
Basic insurance coverage	Social security	205	46.59	75	62.50	80	39.02	360	47.06
	Iran Health Insurance	190	43.18	30	25.00	120	58.54	340	44.44
	Armed forces	0	0	5	4.17	0	0	5	0.65
	Other insurances	45	10.23	10	8.33	5	2.44	60	7.84
	No insurance	0	0	0	0	0	0	0	0
Place of residence	Local (Shiraz)	220	50	60	50	100	48.78	380	49.67
	Non‐local	220	50	60	50	105	51.22	385	50.33

Table 2 shows the number of visits, hospitalization days per year, the costs of each visit, and the average cost of each hospitalization day. According to this table, the amount of each visit in all disease states was equal to $26.19, and the highest number of annual visits was in State 3. Moreover, the average cost of each day of hospitalization was equal to $59.52, and the maximum number of days of hospitalization was 1.

**TABLE 2:** Average costs per visit, number of visits, average cost per hospitalization day, and number of hospitalization days per person‐year.

Cost component	Visit		Admission	
States	Number of visits	Cost per visit ($)	Number of hospitalization days	Average cost per hospitalization day ($)
State 1 (DAS‐28<2.6) Remission	3.37	26.19	4.97	59.52
State 2 (2.6<DAS‐28<3.2) Low	3.26	26.19	4.36	59.52
State 3 (DAS‐28>3.2) Moderate‐to‐severe	3.97	26.19	4.14	59.52
Average costs (average of three states)	3.59	26.19	4.57	59.52

Abbreviation: $, US dollar.

**FIGURE 1:**
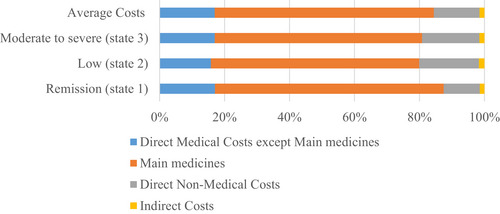
Costs of each patient with RA in different disease states (remission, low, moderate‐to‐severe, and average costs).

Table 3 and Figure 1 show the mean costs of the patients with RA in three disease states. According to this table, the mean DMCs of the patients in remission, low, and moderate‐to‐severe states were $5481.99, $4992.41, and $5437.27, respectively, whereas the DNMCs were $696.05, $1149.80, and $1187.80, respectively, and the ICs were $90.21, $105.79, and $104.36, respectively. Furthermore, the cost of purchasing the main medicines was the highest DMCs in the patients in all three disease states ($4413.00 in remission, $4005.41 in low, and $4293.81 in moderate‐to‐severe states).

**TABLE 3:** Mean annual costs (US dollar [$]) per patient with rheumatoid arthritis (RA) in remission, low, and moderate‐to‐severe states.

States	State 1 (DAS‐28<2.6) Remission		State 2 (2.6<DAS‐28<3.2) Low		State 3 (DAS‐28>3.2) Moderate‐to‐severe		Average costs (average of three states)	
Costs	Mean	%	Mean	%	Mean	%	Mean	%
**Direct medical costs**								
Physicians’ visits	88.26	1.61	85.37	1.71	103.85	1.91	94.02	1.74
Main medicines	4413.00	80.50	4005.41	80.23	4293.81	78.97	4309.17	79.90
Laboratory tests	317.41	5.79	307.53	6.16	373.54	6.87	338.34	6.27
Physiotherapy and other services costs	220.92	4.03	192.71	3.86	247.94	4.56	223.82	4.15
Diagnostic services	146.37	2.67	141.78	2.84	171.82	3.16	155.86	2.89
Hospitalization	296.03	5.40	259.61	5.20	246.31	4.53	271.99	5.04
Total	5481.99	87.46	4992.41	79.90	5437.27	80.80	5393.20	84.42
**Direct non‐medical costs**								
Transportation	404.61	58.13	658.72	57.29	666.32	56.10	514.60	57.24
Accommodation	163.09	23.43	254.91	22.17	269.92	22.72	206.12	22.93
Meals	128.35	18.44	236.17	20.54	251.56	21.18	178.28	19.83
Total	696.05	11.10	1149.80	18.40	1187.80	17.65	899.00	14.07
**Indirect costs**								
Lost income	90.21	1.44	105.79	1.69	104.36	1.55	96.44	1.51
**Total costs**	**6268.24**	**100**	**6248.00**	**100**	**6729.43**	**100**	**6388.64**	**100**

In general, according to the information in Table 2, the total annual costs per patient in remission, low, and moderate‐to‐severe states were $6268.24, $6248.00, and $6729.43, respectively, suggesting that although the difference between the disease states was not great, the costs were generally lower in the low state.

Considering the number of patients in the country estimated using the prevalence rate and based on the mean costs extracted from the results of the present study, the DMCs, DNMCs, and ICs, as well as the estimated economic burden on all the patients with RA in Iran, are presented in Table 4. Thus, the total annual cost of RA patients in Iran was $1,968,337,344 in 2022. The results also showed that DMCs accounted for the majority of the total economic burden of RA in the country (84.42% of the total costs).

**TABLE 4:** Estimation of the total annual costs of rheumatoid arthritis (RA) patients in Iran in 2022 (US dollar [$]).

Number of patients in Iran	DMCs	DNMCs	ICs	COI
308,099	1,661,641,474	276,982,349	29,713,521	1,968,337,344

Abbreviations: COI, cost of illness; DMC, direct medical cost; DNMC, direct non‐medical cost; IC, indirect cost.

## 4 Sensitivity Analysis

The sensitivity analysis outcomes for total cost of illness (COI), ICs, DMCs, and DNMCs are presented in Table 5. In the first scenario (Table 5‐A), where prevalence rates were treated as variable while the properties of costs remained fixed, the analysis yielded the following ranges: DMCs ranged from 1,302,367,642 to 2,065,824,536, DNMCs varied between 217,094,273 and 344,356,434, and ICs fell between 23,288,976 and 36,941,134, with the total COI consequently ranging from 1,542,750,891 to 2,447,122,104.

**TABLE 5:** Sensitivity analyses for direct medical costs (DMCs), direct non‐medical costs (DNMCs), indirect costs (ICs), and total costs of rheumatoid arthritis (RA) patients in Iran in 2022 (US dollar [$]).

Number of patients in Iran		DMCs	DNMCs	ICs	COI
**A: One‐way sensitivity analyses (based on the minimum and maximum number of patients)**					
Lower limit	241,483	1,302,367,642	217,094,273	23,288,976	1,542,750,891
Upper limit	383,042	2,065,824,536	344,356,434	36,941,134	2,447,122,104
**B: One‐way sensitivity analyses (based on the variation of costs)**					
Lower limit	308,099	1,213,910,756	181,810,329	13,870,060	1,409,591,145
Upper limit	308,099	2,109,368,297	372,151,673	45,556,075	2,527,076,045
**C: Two‐way sensitivity analyses (best‐ and worst‐case scenarios)**					
Lower limit	241,483	951,443,566	142,499,988	10,871,128	1,104,814,681
Upper limit	383,042	2,622,457,883	462,675,053	56,637,283	3,141,770,219

Abbreviation: DMC, Direct Medical Costs; DNMC, Direct Non‐Medical Costs IC, Indirect Costs; COI, cost of illness.

The second scenario (Table 5‐B) maintained constant prevalence rates while allowing the properties of costs to vary. Under these conditions, we observed that DMCs ranged from 1,213,910,756 to 2,109,368,297, DNMCs varied between 181,810,329 and 372,151,673, and ICs spanned 13,870,060–45,556,075, resulting in a total COI range of 1,409,591,145–2,527,076,045.

In the comprehensive third scenario (Table 5‐C), where both prevalence rates and the costs of properties were allowed to vary simultaneously, the analysis produced the widest variation in estimates: DMCs ranged from 951,443,566 to 2,622,457,883, DNMCs varied between 142,499,988 and 462,675,053, and ICs spanned 10,871,128–56,637,283, culminating in a total COI range of 1,104,814,681–3,141,770,219.

## 5 Discussion

The costs of treating RA patients have increased rapidly in recent years. The discovery of new medicines over the past few years has made the treatment of this disease more complex and expensive, and although the medicines are very effective, they are associated with significant costs [22, 23]. However, reduced hospital admission rates, better functional status, and lower incidence of disability and absenteeism offset a large portion of pharmaceutical costs [14]. Hence, due to the lack of a comprehensive study on the economic burden of RA in Iran, the present research was carried out to determine the economic burden of RA on the patients referred to the medical centers affiliated with Shiraz University of Medical Sciences in Fars province, Iran, in 2022.

The results of this study indicated that the economic burden of RA was $1,968,337,344 (minimum: 1,104,814,681; maximum: 3,141,770,219). In their study in Italy, Mennini et al. estimated the economic burden of RA at €2 billion ($3.03 billion [adjusted into 2022 international dollars (int$ 2022)]) [24, 25], which is consistent with the results of the present study. In addition, Birnbaum et al. in the United States concluded that the economic burden of RA was $39 billion ($43 billion [adjusted into int$ 2022]) [12, 24], which is inconsistent with the results of the present study. One reason for the inconsistency could be the addition of the cost of premature deaths, as well as the intangible costs, accounting for 80% of the total costs. Furthermore, in a systematic review of RA burden in Latin America, Papadimitropoulos et al. demonstrated that the disease impact in these regions is both significant and comparable to global patterns observed in other geographical areas [26].

The economic burden of RA alone was about 10.07% of the total health expenditures in 2022. The total cost of the health system in Iran was $19.55 billion in 2022, accounting for 6.71% of the GDP in 2022 (Iran's GDP in 2022: $291.36 billion) [27, 28].

Moreover, according to the results of the present study, the mean costs of the disease per patient were $6268.24, $6248.00, $6729.43, and $6388.64 in remission, low, and moderate‐to‐severe states and average costs, respectively. In this regard, the results are consistent with those of the studies by Bali and Singla in India [29], Alghamdi et al. in Saudi Arabia [30], Hu et al. in China [31], Huscher et al. in Germany [14], Malhan et al. in Turkey [32], and Ruof et al. in Germany [33].

The results also showed that DMCs were the greatest total treatment costs, accounting for 87.46%, 79.90%, 80.80%, and 84.42% of the total costs of the disease in remission, low, moderate‐to‐severe, and average costs, respectively, suggesting that DMCs were the most important cost components for the patients with RA. Furthermore, it was found out that the highest share of DMCs was that of purchasing the main medicine (80.50%, 80.23%, 78.97%, and 79.90% of the total DMCs at remission, low, and moderate‐to‐severe states and average costs, respectively), the reason for which could be the high price of the medicines in Iran.

In the current study, the DNMCs accounted for 11.10%, 18.40%, 17.65%, and 14.07% of the total costs of the disease in remission, low, and moderate‐to‐severe states, and average costs, respectively. Hsieh et al. conducted a systematic research examining 72 studies carried out from 2000 to 2022 on the economic burden of RA and stated that the cost of the medicines was a major component of direct costs, and the costs increased over time [23]. Alghamdi et al., in their cost analysis of 400 RA patients in Saudi Arabia, identified biologic agents as the primary cost driver (84% of total expenditures), followed by laboratory/diagnostic tests (5%) and outpatient visits (3%) [30]. In a study in China examining the burden of arthritis from a societal perspective, Hu et al. [31] concluded that the mean direct costs were $2559.06 ± 1917.21 ($790.64 ± 592.28 [adjusted into int$ 2022 [24]]) per patient a year, and the costs of purchasing the medicines accounted for over 50% of the total costs $1898.15 ± 1283.89 ($586.41 ± 396.681 [adjusted into int$ 2022 [24]]). On the other hand, age and income were significantly associated with indirect and intangible costs [31]. In a study on 689 patients in Turkey, Hamuryudan et al. [34] examined the direct costs and ICs of arthritis and showed that direct costs and ICs account for 64% and 36% of the total costs, respectively (annual direct costs and ICs were €4954 ($9089.68) and €2802 ($5141.16 [adjusted into int$ 2022 [24]]) per year). Medicine costs accounted for over 50% of direct costs (average €2777 ($5095.29 [adjusted into int$ 2022 [24]])), second to which were the RA‐related consulting costs (average, €1600 ($2935.71 [adjusted into int$ 2022])) [24, 34]. Michaud et al. in the United States examined the direct costs of RA and showed that the mean total cost of annual direct medical care in 2001 was $9519 ($2551.40 [adjusted into int$ 2022 [24]]) per RA patient [35]. The results of these studies are consistent with those of the present study.

However, Naqvi et al. conducted a study on 358 patients in Pakistan and indicated that the total annual costs of arthritis were $891.83 ($873.58 [adjusted into int$ 2022 [24]]), of which physiotherapy costs accounted for the highest percentage of the total annual costs and medicine costs accounted for only 7.1% of costs [36], which is not consistent with the present study. Catay et al. [37] conducted a study on 165 patients in Argentina and found that the mean DMCs and DNMCs accounted for 60% and 7% of the total costs, respectively, and ICs were 23% of total costs. Hospitalization accounted for 73% of the total DMCs, whereas medicines and outpatient procedures accounted for 16% and 8% of the total DMCs, respectively. The results differ from those of the present study, the reason for which could be the high costs of hospitalization in that country [37].

The present study also showed that ICs accounted for the lowest total treatment costs were 1.44%, 1.69%, 1.55%, and 1.51% of the total costs in remission, low, moderate‐to‐severe states and average costs, respectively. The results of this study are consistent with those of the studies by Grega and Kolář in the Slovak Republic [38], Xu et al. in China [39], and Osiri et al. in Thailand [40]. However, in a systematic review, Hsieh et al. [23] showed that ICs were primarily related to absenteeism and disability, accounting for 39%–86% of the total costs. Moreover, the ICs reported were highly sensitive to the estimation approach [23]. This is different from our study, one of the reasons for which could be the difference in the approaches used to estimate the costs. According to Zhu et al., who conducted a study on RA patients in Hong Kong, the mean total costs of RA were $9286 ($10,633 [adjusted into int$ 2022 [24]]) per patient, of which more than 60% was related to ICs due to the productivity loss [41]. This might be due to the higher wages in Hong Kong. In their study, Filipovic et al. [42] showed that productivity reduction and related costs varied in different studies, but all studies showed that RA was associated with significant direct costs. They also argued that economic analyses that eliminated ICs underestimated the full economic impact of RA. This is inconsistent with the present study, in which the ICs had a low share. Nevertheless, as mentioned, it is generally better to include ICs in the analyses to provide a more accurate estimate of the disease. However, it was stated that the methods used to calculate productivity loss had a significant impact on the results of IC's analysis and that they should be carefully selected when designing such studies [42]. This could be one of the reasons why the ICs were different in this study.

### 5.1 Study Limitations

One limitation of the present study was the self‐report of the patients or their companions about DNMCs and ICs, because they were more likely to forget or approximate some of the costs. In addition, due to the lack of accurate evidence on the number of RA patients in Iran, the opinions of some of the best experts in this field were used in the present study. It is worth mentioning that intangible costs were not calculated in this study due to the inability to measure them accurately.

### 5.2 Policy Implications

Regarding the results, to decrease the RA economic burden, the high share of medicine costs, and the large amount of the cost of importing medicine from other countries, it is proposed to provide a context for producing these medicines in the country.

Besides, given the high share of the transportation costs, it is suggested to decrease these costs by equitable and proper distribution of physicians, developing the home care center, and expanding telemedicine services.

## 6 Conclusions

In general, the results showed that the economic burden of RA was about $1,968,337,344 (minimum: 1,104,814,681; maximum: 3,141,770,219) in 2022, accounting for about 0.7% of the GDP in 2022. In addition, RA could impose a heavy economic burden on the health care system, insurance system, and the patients themselves due to its relatively high prevalence in Iran and the world, being chronic, as well as needing lifelong treatment and the treatment costs. According to the results of the present study, DMCs accounted for the highest costs, the largest share of which were the costs of medicines.

According to the obtained results, and in order to reduce the economic burden of RA, it is suggested that health managers and policy makers provide necessary facilities for the production of these medicines in the country because of the high medicine costs and high prices of foreign medicines.

## Author Contributions

**Mehdi Rezaee**: conceptualization, methodology, data curation, software, supervision, validation, writing – review and editing. **Farhad Lotfi**: conceptualization, methodology, data curation, software, supervision, validation, roles/writing – original draft. **Ahmad Gholami**: conceptualization, data curation, methodology, supervision, validation, writing – review and editing. **Jassem Azizpoor**: data curation, methodology, software, validation, writing – review and editing. **Elham Aflaki**: conceptualization, methodology, supervision, validation, writing – review and editing. **Afsaneh Vazin**: conceptualization, methodology, validation, writing – review and editing. **Khosro Keshavarz**: conceptualization, data curation, methodology, software, project administration, investigation, supervision, validation, writing – review and editing.

## Ethics Statement

All data were collected and handled in accordance with the relevant privacy protection guidelines. This study was approved by Shiraz University of Medical Sciences with the Grant No. 97‐01‐07‐18106 and ethical code IR.SUMS.REC.1399.100. Verbal consent was obtained from respondents who completed a questionnaire anonymously, and their response expressed their willingness to participate. Written consent was obtained from participants for the cognitive interviews.

## Consent

All participants completed a consent form, stating that they were well‐informed about the content of questionnaires and that they agreed to the publication of anonymized data.

## Conflicts of Interest

The authors declare no conflicts of interest.

## Data Availability

The datasets generated and analyzed during the current study are not publicly available because they contain information that could compromise the privacy of research participants but are available from the corresponding author upon reasonable request.
